# Quantitative and longitudinal assessment of human placental inflammation using diffusion basis spectrum imaging

**DOI:** 10.1038/s44294-024-00049-5

**Published:** 2025-01-03

**Authors:** Zhexian Sun, Wenjie Wu, Zezhen Xiang, Hansong Gao, Weina Ju, Cherilyn Uhm, Ian S. Hagemann, Pamela K. Woodard, Nanbert Zhong, Alison G. Cahill, Qing Wang, Yong Wang

**Affiliations:** 1 Department of Biomedical Engineering, Washington University in St Louis, St Louis, MO USA; 2 Department of Obstetrics and Gynecology, Washington University School of Medicine, Washington University in St Louis, St Louis, MO USA; 3 Department of Electrical & Systems Engineering, Washington University in St Louis, St Louis, MO USA; 4 New York State Institute for Basic Research in Developmental Disabilities, Staten Island, NY USA; 5 Department of Pathology & Immunology, Washington University School of Medicine, Washington University in St Louis, St Louis, MO USA; 6 Mallinckrodt Institute of Radiology, Washington University School of Medicine, Washington University in St Louis, St Louis, MO USA; 7 Department of Women’s Health, Dell Medical School, University of Texas, Austin, TX USA

**Keywords:** Reproductive disorders, Reproductive disorders

## Abstract

Besides exchanging nutrients, gases, and wastes, placenta is an intermediary between maternal and fetal immune systems. However, no method exists to safely image and monitor placental inflammation during pregnancy. We customized a Magnetic Resonance Imaging (MRI) method, diffusion basis spectrum imaging (DBSI), to measure immune cells in placenta. We validated placental DBSI in simulations and ex-vivo specimens, then applied it to 202 MRI scans from 82 patients whose placentas were classified as non-inflammation (*n* = 70) or inflammation (*n* = 12). Our method imaged the 3D distribution of immune cells, revealing significantly greater infiltration in the inflammation placentas from early (2.8% ± 0.7% vs. 4.8% ± 0.65%, *p* < 0.01) to late pregnancy (4.75% ± 0.9% vs. 7.25% ± 2.13%, *p* < 0.01). DBSI detects elevated immune cell infiltration associated with placental inflammation and enables non-invasive imaging of placental inflammation, offering early detection and monitoring throughout pregnancy, facilitating personalized care and potentially improving pregnancy outcomes without ionizing radiation.

## Introduction

A healthy placenta is essential for fetal well-being because it delivers oxygen and nutrients and removes carbon dioxide and other waste products^1^. The placenta also functions as an intermediary between the maternal immune system and the fetus and protects the fetus from pathogens^2^. Recent placental research has focused on diverse aspects of placental function, maternal-fetal health, and potential therapeutic applications. Studies have explored the impact of environmental factors on placental health, investigated the levels of non-essential trace metals and their effects on placental function^3^. Research has also emphasized the critical role of timely medical intervention in placental complications, which comes from a study linking maternal late hospital arrival with adverse fetal outcomes in placental abruption cases^4^.

In a normal human pregnancy, the intrauterine immune response starts as soon as implantation^5^. The placental immune response evolves across the gestation, and each trimester is characterized by a unique inflammation environment^6^. The accumulation of immune response could trigger the labor onset^7^. Several types of immune cells function in the placenta^8,9^. In a healthy placenta, uterine or decidual natural killer (NK) cells, maternal/fetal macrophages, dendritic cells, and regulatory T cells participate in spiral artery remodeling and trophoblast invasion. In pathological scenarios, additional immune cells often infiltrate the placenta. For example, neutrophils enter the placenta in cases of chorioamnionitis, chorionic vasculitis, and acute villitis^10^; eosinophils enter the placenta in cases of intervillous thrombosis, cysts, and chorionic vasculitis^11^; basophils enter the placenta in cases of acute placental infarction^11^; increased macrophages enter the placenta in cases of listeria, tuberculosis, syphilis, acute atherosis, and villitis of unknown etiology^12–14^; and CD4 + T cells significantly increase in cases of villitis of unknown etiology^15^. However, most of our knowledge of these processes in humans comes from immunohistochemistry of the ex vivo placenta after delivery. To longitudinally monitor immune infiltration of the placenta in pregnancy, we need a safe, non-invasive, and accurate imaging modality.

Current imaging techniques for monitoring placental conditions, such as ultrasound and computed tomography (CT), have significant limitations. Ultrasound is widely used for placental assessment due to its cost-effectiveness and real-time imaging capabilities. However, its accuracy can be compromised by factors like maternal obesity and bladder fullness. Additionally, ultrasound resolution diminishes in the later stages of pregnancy, making image interpretation challenging due to low contrast. Moreover, ultrasound is not sensitive to detecting placental inflammation. While CT can be valuable for assessing trauma during pregnancy, its use is restricted by the risks associated with ionizing radiation exposure, limiting its broader application for routine placental monitoring. As an emerging clinical imaging tool in the field of Obstetrics and Gynecology, MRI forms high-quality and multi-contrast 3D images using water molecules, which are naturally abundant in the human body. Therefore, MRI can be employed repeatedly in longitudinal studies to assess human pregnancy. Latest advancements in MRI, such as diffusion-relaxation MRI and T2*-weighted MRI, have become an important tool in placental research. Diffusion-relaxation MRI have been used to detect altered placental development in pregnancies affected by congenital heart disease (CHD). A study found significant differences in placental microstructure and perfusion between CHD-affected pregnancies and controls, particularly after 30 weeks’ gestation^16^. T2*-weighted placental MRI has been investigated as both a basic research tool and a potential clinical test for placental dysfunction. This imaging technique shows promise in directly reflecting placental function, which could significantly improve antenatal screening for placental dysfunction^17^. Especially, diffusion MRI (dMRI) enables the researcher to image and quantify the great complexity and heterogeneity in human placental microstructure^18^. In hemodynamic studies, dMRI enabled the exploration of the blood flow in the healthy placenta^19^ and the mal-perfusion in pre-eclampsia^20^. dMRI was also used to track the myometrium alternation induced by placenta accrete^21^. In addition, merging dMRI with other MR contrasts brings new insight into the compartment-wise oxygenation property^22^.

Although dMRI was successfully applied to detect inflammation in brain and other organs^23–25^, its placenta application has not yet yield imaging biomarkers specific to placental inflammation. To overcome a similar challenge of measuring neuro-inflammation in vivo in human brain, DBSI was successfully developed^26,27^. This advanced dMRI method is able to disentangle the highly restricted intracellular diffusion signal associated with cellularity from the diffusion associated with other microstructural components. DBSI has been extensively validated in the brain to detect increased cellularity during inflammation demyelination^26^ and other forms of neuroinflammation in tumors^28^. Here, we customized DBSI based on the unique placental microenvironment to image immune cell infiltration in the human placenta. We performed ex vivo and simulation experiments to develop and validate the method and then used it for in vivo longitudinal analysis of the placenta. We found that our in vivo placental DBSI method reveals an increasing immune response after the first trimester, particularly in the placenta tissues compartment. We also discovered greater immune cellularity in placentas with pathology-defined signs of inflammation than in placentas without signs of inflammation at as early as 11–13 weeks’ gestation.

## Results

### Demographic information

A total of 91 patients were enrolled in the study, and 239 MRI scans were scheduled, ranging from 12 to 38 weeks. MRI scans were scheduled on the same day as the patients’ regular obstetric visits at approximately 12, 20, 32, and 36 weeks’ gestation. A few patients were dual enrolled in other studies and thus underwent additional MRI scans. 202 scans from 82 patients were analyzed after excluding unfinished scans and poor-quality scans which are not sufficient to be analyzed by DBSI. At delivery, placentas from all participants were collected for pathologic and single-nucleus RNA sequencing examination. We used the pathological diagnosis to divide the cohort into two groups. The placentas of 12 patients were defined as the inflammation group (Table 1, Supplemantary Table 1). The placentas from 70 patients did not have any of these diagnoses and were defined as the non-inflammation group. There were no significant differences in age (29.17 ± 4.15 years vs. 29.28 ± 4.98 years) or body mass index at delivery (27.42 ± 5.89 kg/m^2^ vs. 28.04 ± 5.79 kg/m^2^) between the inflammation and non-inflammation groups. A subset of the non-inflammation group (50 scans from 17 patients) who have additional T2* scans was used to conduct intra-placental segmentation. To perform ex-vivo validation, we obtained the ex-vivo MRI and immunohistochemistry (IHC) from five placenta after delivery. Among the five IHCs, we built 3D geometry on 400 0.25 × 0.25 mm^2^ regions and conducted a Monte Carlo simulation.

**Table 1:** Demographic and clinical information

	Age	BMI	Gestational age	Pathology
Non-inflammation group (*n* = 70)	29.28 ± 4.98	28.04 ± 5.79	266.58 ± 20.62	
Inflammation group (*n* = 12)	29.17 ± 4.15	27.42 ± 5.89	265.83 ± 18.12	
Pt #1	36	23.16	281	Multifocal villitis with associated intervillositis Fetal membrane with pigmented amniotic macrophages.
Pt #7	31	28.34	274	Fetal membranes with numerous pigment-laden amniotic macrophages
Pt #14	20	18.54	280	Fetal membrane with moderate acute chorioamnionitis Trivascular cord with modera funisitis
Pt #35	27	25.97	263	Acute chorionitis Trivascular cord with umbilical arteritis
Pt #42	26	33.80	278	Fetal membranes with numerous pigmented amnionic macrophages Chronic lymphohistiocytic villitis, non-necrotizing
Pt #44	29	28.30	277	Chronic lymphohistiocytic villitis Increased perivillous fiberinoid deposition, approximately 10% of volume
Pt #48	32	22.04	260	Chronic lymphohistiocytic villitis, mild/focal, non-necrotizing
Pt #50	29	35.98	243	Acute chorionitis
Pt #60	27	20.45	220	Fetal membrane with acute chorioamnionitis
Pt #109	34	25.14	264	Incidental septal cyst (2 cm in greatest dimension)
Pt #112	28	32.01	273	Intervillous fibrin Peripheral placental cyst with 0.9 cm greatest dimension.
Pt #116	31	35.35	277	Fetal membranes with pigmented amniotic macrophages Mild acute chorioamnionitis
*p*-value	0.88	0.76	0.90	

### Development and validation of a whole placenta DBSI method to identify immune cells

**Fig. 1:**
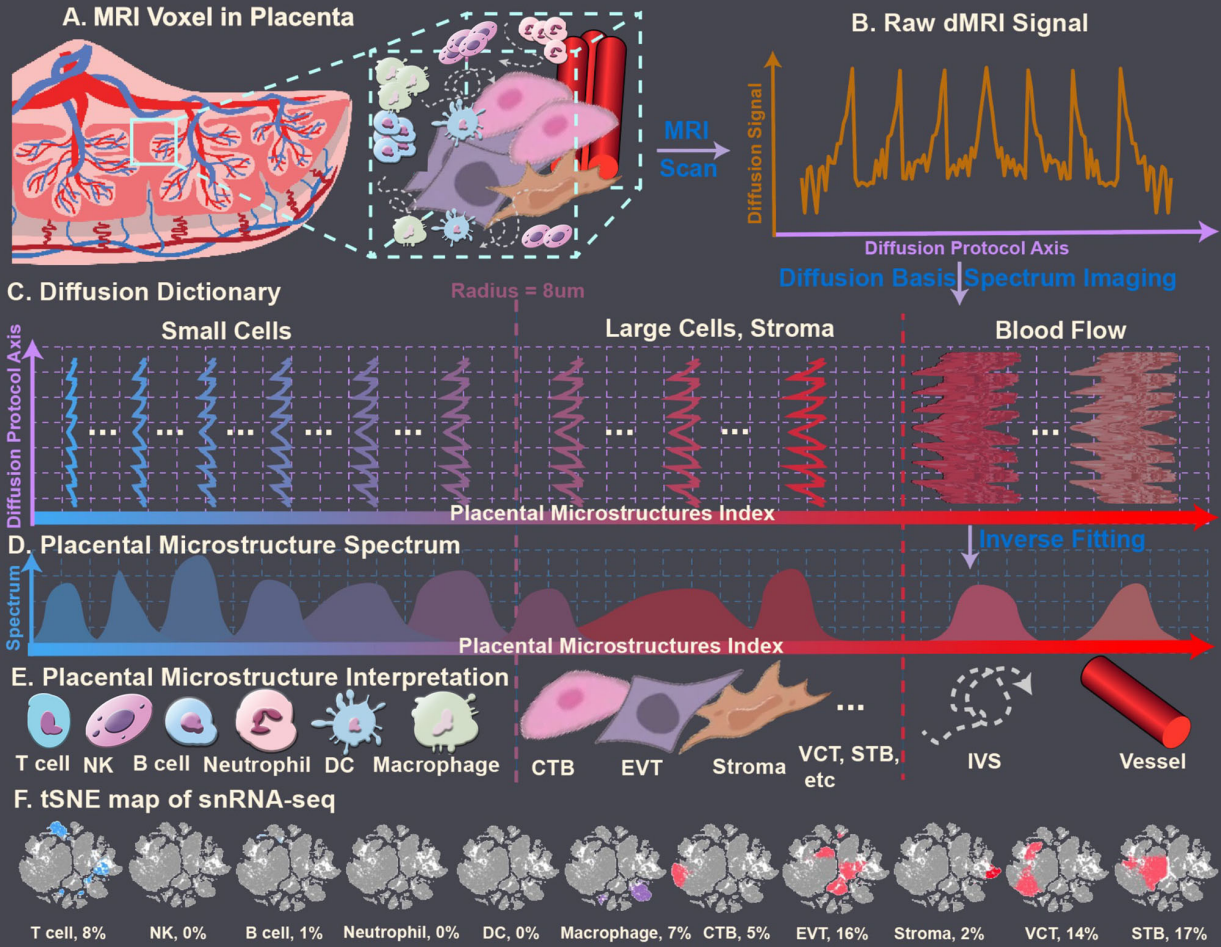
Schematic demonstration of diffusion basis spectrum imaging (DBSI) methodology. **A** Schematic of a Magnetic Resonance Imaging (MRI) voxel that contains several microstructures. **B** Schematic of measured diffusion signals from the voxel. **C** Diffusion dictionary assembled from prior knowledge. **D** DBSI-derived spectrum. **E** The dictionary corresponds to placental cells of different radii: T cells, 2–3 µm; natural killer (NK) cells, 3–4 µm; B cells, 4–5 µm; neutrophils, 6–7 µm; dendritic cells (DC), 7–10 µm; macrophages, 10–11 µm; cytotrophoblasts (CTB), syncytiotrophoblast (STB), extravillous trophoblasts (EVT), and decidua stromal (dS) cells, and other placental resident cells, >10 µm. **F** t-distributed stochastic neighbor embedding (tSNE) map of variant types of placental cells and their percentage, which were annotated (and labeled from blue color to red color, correlated to Diffusion Dictionary) by single-cell RNA sequencing.

In DBSI, “dictionaries” are used to disentangle diffusion characteristics of water molecules in and around multiple cellular components within an imaging voxel (Fig. 1). For this study, we were most interested in distinguishing immune cells from other cells in the placenta. To do so, we took advantage of the large difference in the sizes of placental cells. Using the placenta collected in this study, single-nucleus RNA sequencing (snRNA-seq) was conducted to determine the composition of cell types in the placenta (Fig. 1F).

The decidua is largely composed of round decidual and trophoblast giant cells, and the chorionic plate is mainly composed of fetal blood vessels embedded in fetal stromal tissue, lined by trophoblastic cells. Those decidual and trophoblast cells are usually over 40 µm in the largest dimension, and many have irregular shapes. The chorionic villi are mainly composed of cytotrophoblasts, which have a radius over 20 µm, and syncytiotrophoblasts, which are formed by the fusion of many cytotrophoblasts. In contrast, immune cells are generally less than 8 µm in radius. Thus, we conducted a Monte-Carlo simulation on cell structures with a radius of 8 µm (Supplementary Material 3). The computed cell apparent diffusion coefficient (ADC) was 0.6 mm^2^/msec. Therefore, our DBSI analysis method extracted placental immune cells from the isotropic spectra by using ADC values between 0.01 and 0.6 mm^2^/msec (Fig. 1).

**Fig. 2:**
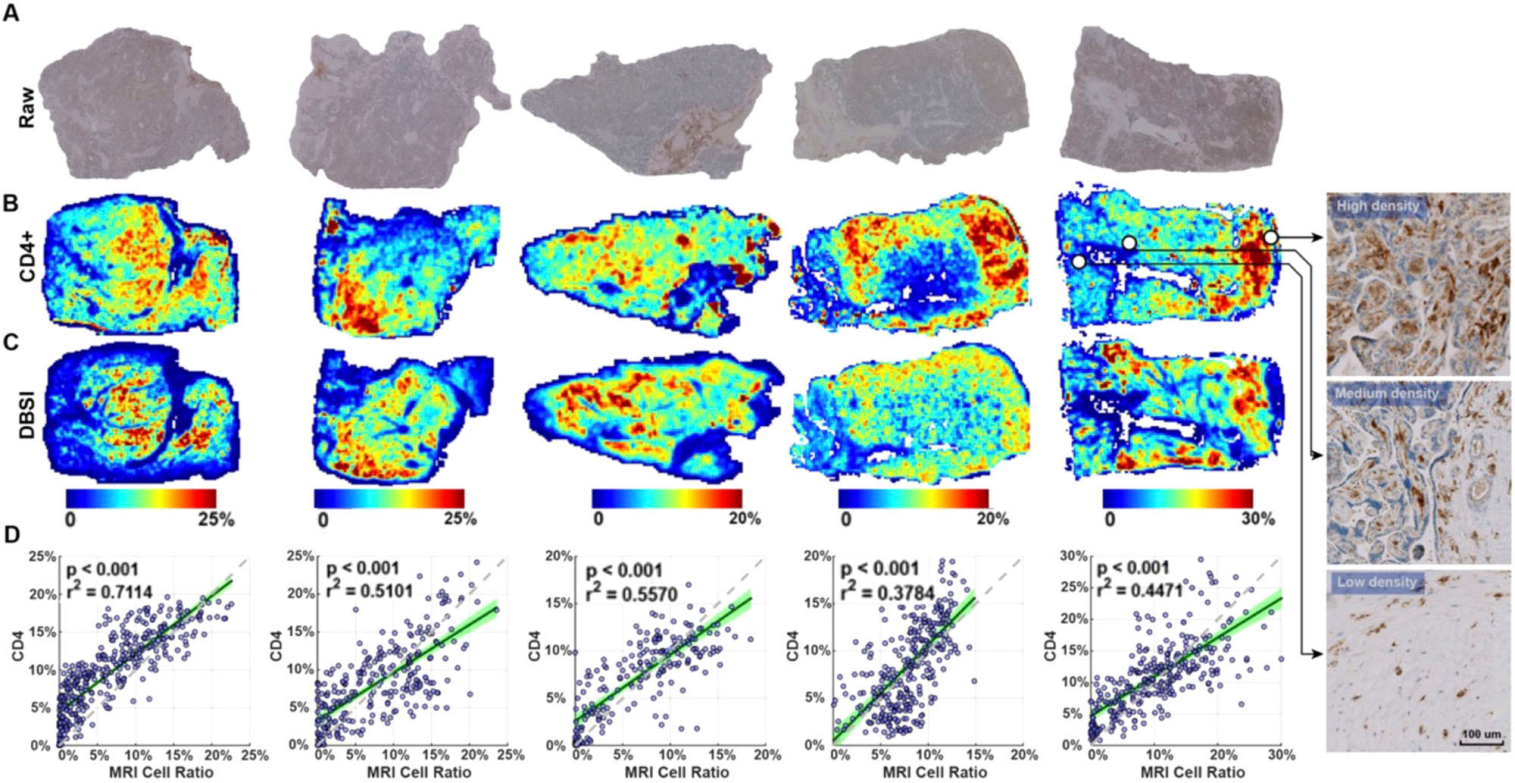
Correlation between DBSI- and immunohistochemistry-derived immune cell densities. **A** Raw immunohistochemistry (IHC) images of CD4 staining. **B** Positive stain area ratio on CD4 IHC, registered to the corresponding DBSI image. Images at the far right are high-magnification views of indicated regions in raw CD4 staining images. **C** DBSI-estimated immune cell density. The IHC- and DBSI-derived maps share the same heatmap keys. **D** Correlation between the DBSI- and IHC-derived immune cell densities. Each point represents the mean value in a 5-by-5 pixel patch in (**A**) and (**B**). The 95% confidence intervals of the linear regressions are shaded green. The dashed line has a slope of 1 and is shown as a reference.

To determine whether DBSI could be used to assess immune cell infiltration in the placenta accurately, we performed ex vivo validation. We obtained samples from five placentas after delivery, embedded them in agar, and performed ex vivo MRI. We then bisected the samples, fixed them, embedded them in paraffin, cut sections, and performed IHC with an anti-CD4 antibody (Fig. 2A). We quantified the CD4-positive area and used landmarks to register the IHC-derived immune cell density maps (Fig. 2B) with DBSI-derived immune cell density maps (Fig. 2C). Qualitatively, we found similar patterns in all samples in the DBSI-derived and IHC-derived maps (Fig. 2B, C). Linear regressions of the data from all five samples showed significant correlations (r^2^ between 0.38 and 0.71, *p* < 0.001). Moreover, the estimated slopes from the linear regressions were all close to 1 (Fig. 2D).

**Fig. 3:**
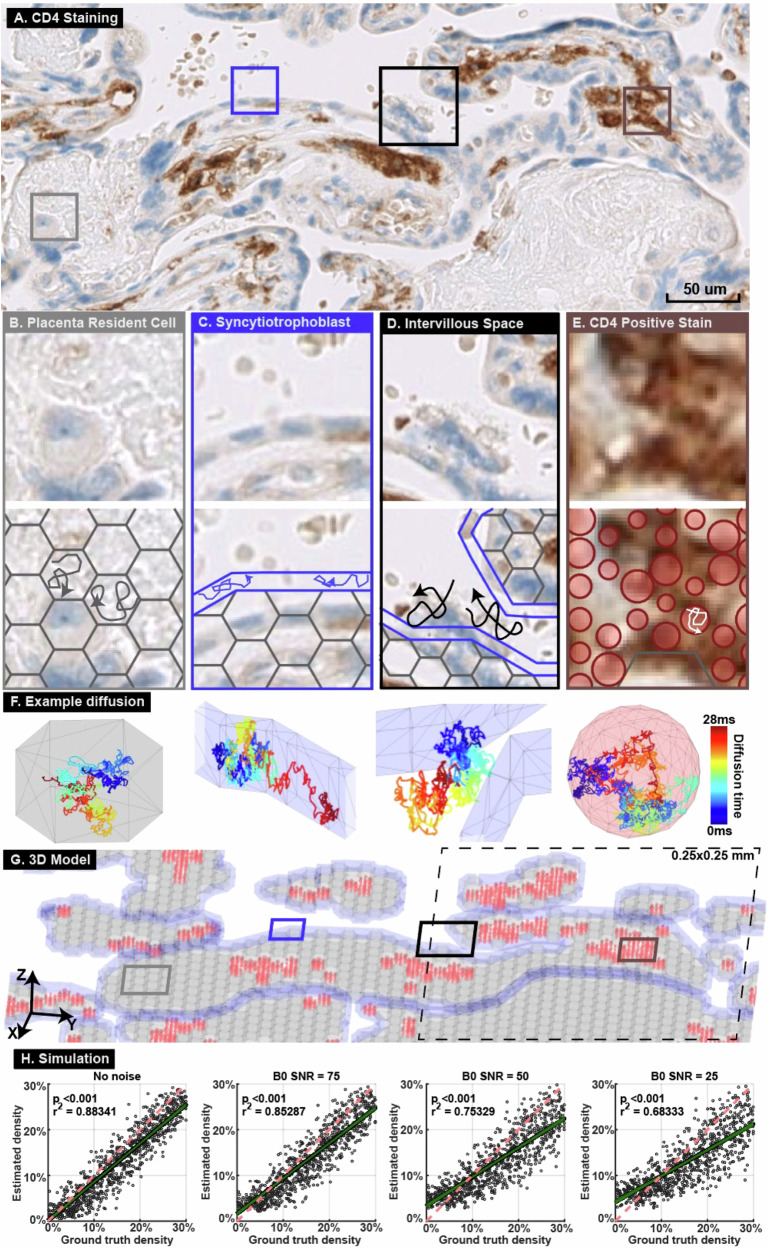
Monte Carlo simulation of diffusion MRI based on histology images. **A** CD4 staining of a placenta specimen. Four representative regions were selected (boxes). **B** Hexagonal prisms were used to model placental resident cells. **C** A tube surrounding the entire villus was used to model the syncytiotrophoblast layer. **D** Water molecules were placed outside the villous model to simulate diffusion in the intervillous space. **E** Spheres of radius 4–8 µm were placed where CD4 positive stain was detected to represent immune cells. **F** Diffusion trajectories from the example random seeds in the four structures. **G** Built a 3D model based on the histology image in (**A**). The entire 3D model covers a 5 × 5 mm region and is the input for the Monte Carlo simulation. The model was divided into 400 0.25 × 0.25 mm regions, one of which is illustrated in the dashed-line box. Hexagonal prisms representing resident cells are marked grey, models representing syncytiotrophoblasts are marked blue, and spheres representing immune cells are marked red. **H** Diffusion MRI signals were simulated in 400 0.25 × 0.25 mm regions with various signal-to-noise ratios. The simulated MRI- and IHC-derived immune cell densities were compared by linear regression. The green line shows the linear regression and 95% confidence range. The red dashed line has slope of 1 and is shown as a reference.

To assess our method’s sensitivity and noise resistance, we built a Monte Carlo simulated three-dimensional microstructure geometric model derived from histology images collected in this study (Fig. 3A–G, Supplementary Material 4). We then simulated diffusion MRI signals with different signal-to-noise ratios consistent with the ranges observed in ex vivo and in vivo MRI data (58.6 ± 7.9 and 37.4 ± 3.7, respectively). We simulated 400 0.25 × 0.25 mm regions on histology images. There was a significant linear correlation between the simulated MRI-derived cell density and the IHC-derived cell density under signal-to-noise ratios of 75 to 25 (r^2^ between 0.68 and 0.89, *p* < 0.001, Fig. 3H).

### DBSI analysis of placentas in vivo

**Fig. 4:**
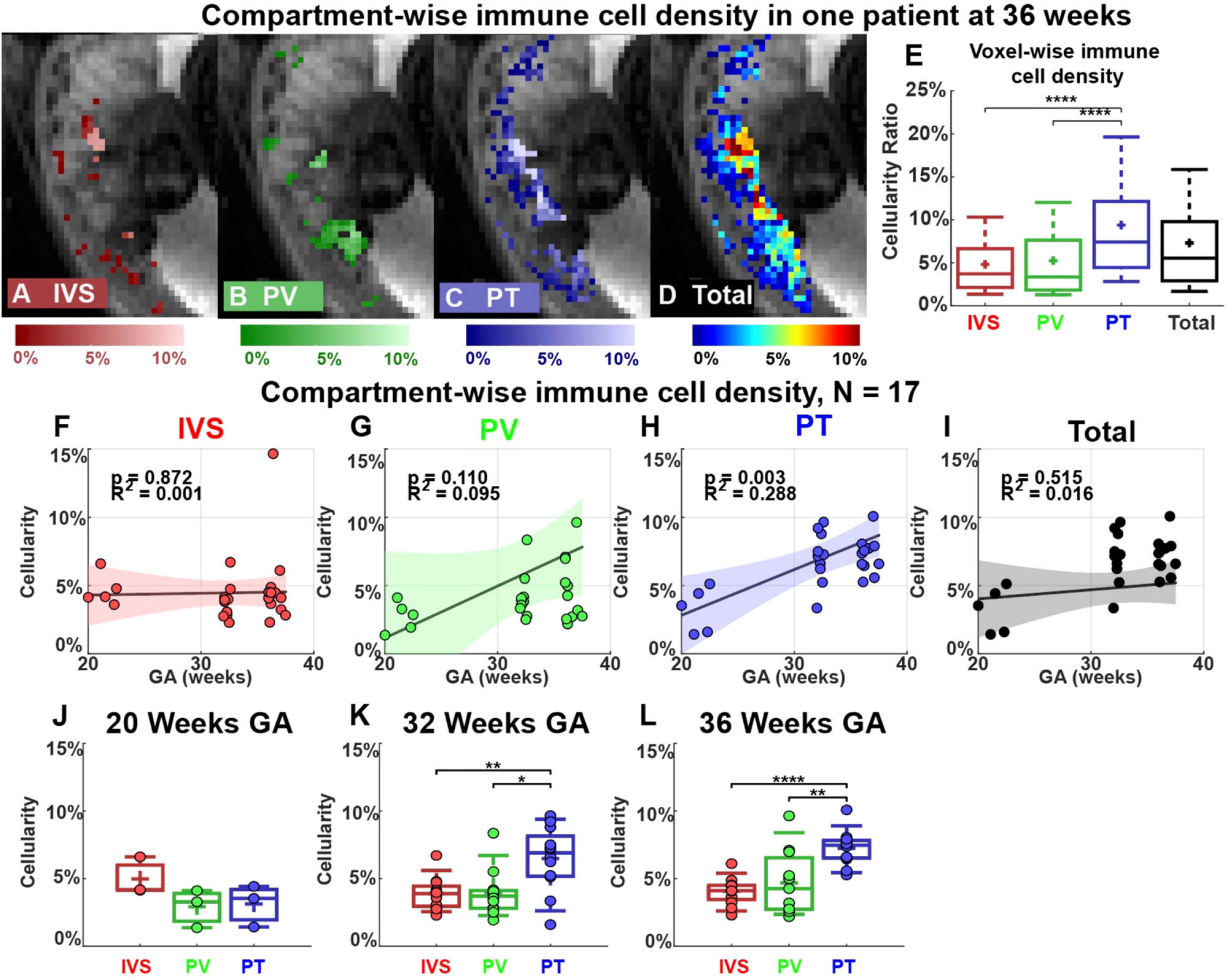
Cellularity ratio in each placental compartment. **A**–**D** Representative cellularity maps in the intervillous space (IVS), placental vessels (PV), placental tissue (PT), and total placenta are illustrated in heat maps. **E** Boxplots of voxel-wise cellularity ratio in the representative placental regions in (**A**–**D**). **F**–**I** Linear regressions of the mean cellularity ratios in each compartment and entire placenta. The colored region indicates a 95% confidence interval. **J**–**L** Comparison of mean cellularity in each visit (20/32/36 weeks). In the box plots, the horizontal line indicates the median, the + indicates the mean, the edges of the box indicate the 25th and 75th percentiles and the whiskers indicate 95% confidence intervals. *p < 0.05, **p < 0.01, ****p < 0.0001 by Wilcoxon Rank-Sum test.

We first used DBSI to identify the placental regions that had the most immune cell infiltration. To do so, we used our previously developed and published intra-placental segmentation method based on T2* and diffusion MRI to divide the placenta into three major compartments: intervillous space, placental vessels, and placental tissue^29^. We combined this method with the placental DBSI analysis on 50 MRI scans from 17 patients in the non-inflammation group who have both T2* and diffusion MRI scans. Figure 4A–D shows images from a representative patient (29 years old, viable male infant delivered at 39 weeks and 4 days) imaged at 36 weeks’ gestation. The highest voxel-wise cellularity ratio was in the placental tissue region (Fig. 4C, E). We then combined all the data from the 17 patients and analyzed them in two ways. First, we used linear regression to examine the cellularity ratio in each placental region at multiple time points in pregnancy (Fig. 4F–I). This analysis revealed that cellularity was significantly greater in the placental tissue region late in pregnancy than early in pregnancy (r^2^ = 0.288, *p* = 0.003, Fig. 4H). Second, we compared cellularity between the compartments at each time point (Fig. 4J–L). This analysis showed that cellularity was significantly greater in the placental tissue region than in the placental vessels or intervillous space regions at 32 weeks’ gestation (*p* < 0.05) and 36 weeks’ gestation (*p* < 0.01) (Fig. 4K, L).

**Fig. 5:**
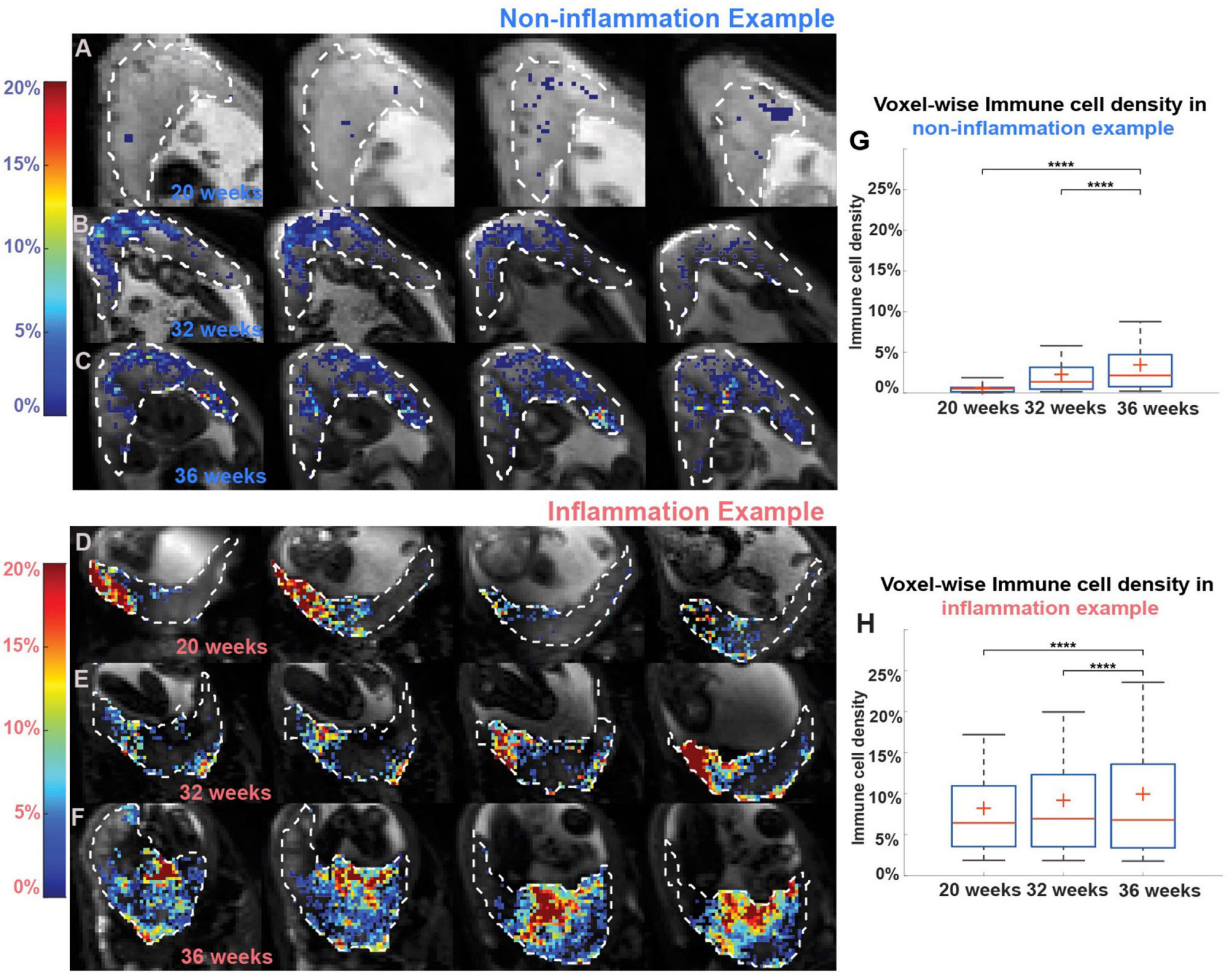
Longitudinal progress of placental cellularity in two representative placentas. **A**–**F** Placental cellularity map at 20, 32, and 36 weeks’ gestation from (**A**–**C**), a patient in the non-inflammation group, and (**D**–**F**), a patient in the inflammation group. Background gray scaled images are T2WI. Cellularity ratios >0.01 are depicted in heat maps. **G**, **H** Boxplots of voxel-wise placental cellularity ratio across three-time points in the two patients. In the box plots, the horizontal line indicates the median, the + indicates the mean, the edges of the box indicate the 25th and 75th percentiles and the whiskers indicate 95% confidence intervals. ****p < 0.0001 by Wilcoxon Rank-Sum test.

Next, we compared our placental DBSI data from the two groups of placentas: non-inflammation and inflammation. To visualize and compare the immune cell density in the two groups, we plotted the color-coded immune cell density maps on top of the anatomical reference T2 weighted images. Figure 5A–F shows four axial views of different MRI slices from one representative non-inflammation placenta and from one representative inflammation placenta (patient #109, see Supplementary Table 1 for details) at 20, 32, and 36 weeks’ gestation. Qualitatively, immune cell density appeared higher in the inflammation placenta (Fig. 5D–F) than in the non-inflammation placenta (Fig. 5A–C). In both placentas, voxel-wise cell density increased over pregnancy (Fig. 5G, H).

**Fig. 6:**
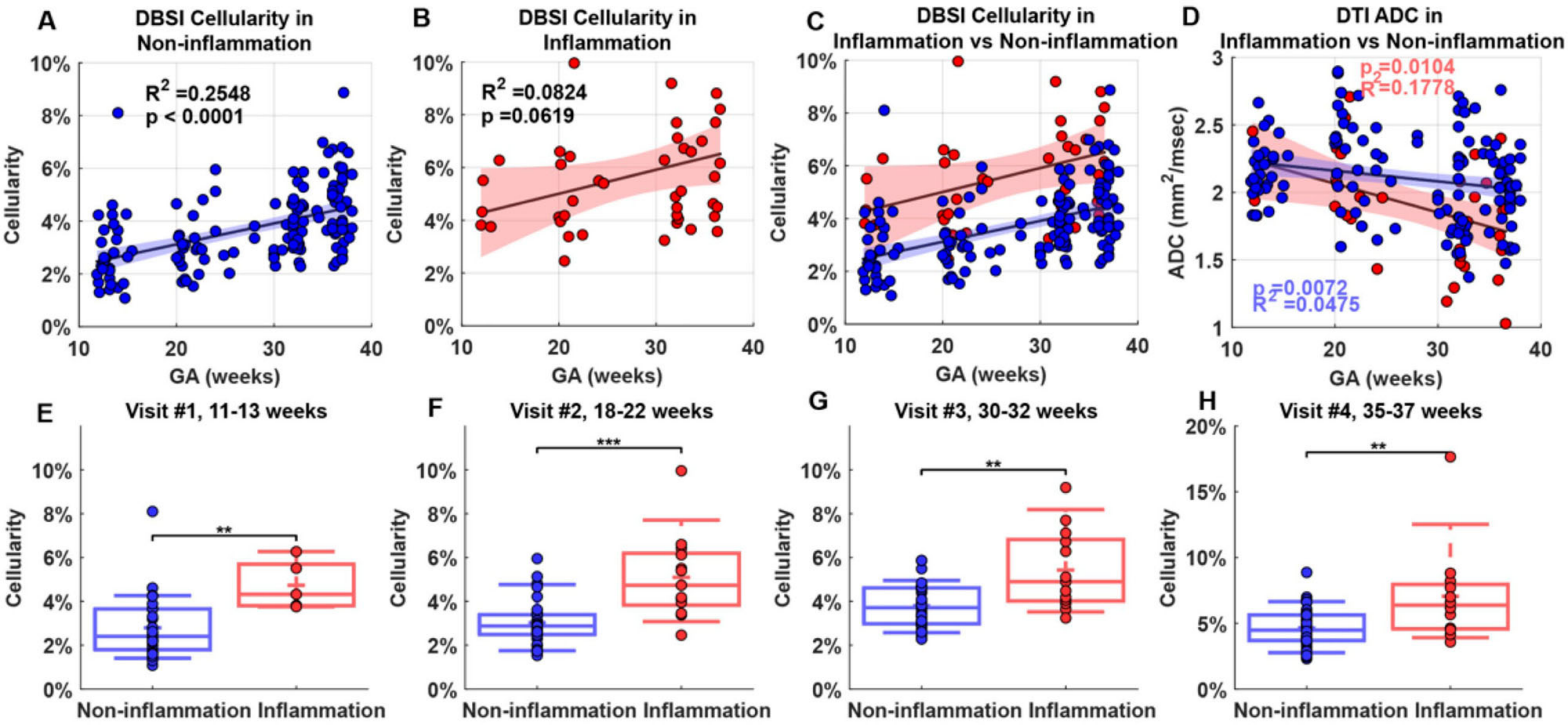
Longitudinal placental mean cellularity. **A**, **B** Mean cellularity ratio in the (**A**) non-inflammation group and (**B**) inflammation group. **C** Combined plot of (**A**) and (**B**). **D** Mean placental apparent diffusion coefficient (ADC) in both groups; blue indicates non-inflammation, and red indicates inflammation group. In (**A**–**D**), colored regions indicate 95% confidence intervals. **E**–**H** Comparison of cellularity ratio between the two groups at the indicated weeks’ gestation. In the box plots, the horizontal line indicates the median, the + indicates the mean, the edges of the box indicate the 25th and 75th percentiles and the whiskers indicate 95% confidence intervals. **p < 0.01; ***p < 0.001 by Wilcoxon Rank-Sum test.

We asked whether there were group-wise differences in immune cellularity between non-inflammation and inflammation placentas. We performed linear regressions on the mean placental cellularity ratios for each of the two groups (Fig. 6A–D). Cellularity was significantly greater later in pregnancy than earlier in the non-inflammation group (r^2^ = 0.2548, *p* < 0.0001) but not in the inflammation group (r^2^ = 0.0824, *p* = 0.0619) (Fig. 6A, B). Throughout pregnancy, the cellularity ratio was significantly higher in the placentas from the inflammation group than in the placentas from the non-inflammation group (Fig. 6C). This difference was significant at each of the four time points (*p* < 0.01) (Fig. 6E–H).

Finally, to compare DBSI to another MRI method, diffusion tensor imaging, we examined mean ADC in non-inflammation and inflammation placentas. Diffusion tensor imaging-derived ADC values did not differ between non-inflammation and inflammation placentas at any time point (Supplementary Material 5). Moreover, in a linear regression of ADC values, the non-inflammation and inflammation groups did not significantly differ from each other (Fig. 6D). This finding suggests that diffusion tensor imaging failed to distinguish between the non-inflammation and inflammation groups compared to DBSI.

## Discussion

Herein, we developed and validated a DBSI method to image and quantify immune cell density in the human placenta across gestation. Additionally, we used our method to reveal placental differences between non-inflammation and inflammation cases, as defined by the placental pathology reports after delivery. Our method could detect significant differences in immune infiltration as early as 11–13 weeks’ gestation. With further clinical validation, this DBSI method could be used clinically to identify pregnant patients with excess placental inflammation. Early detection would allow for timely interventions, such as enhanced monitoring or anti-inflammatory treatments, potentially preventing complications like preeclampsia or preterm birth. This approach could enable more personalized management of high-risk pregnancies, significantly improving both maternal and fetal outcomes.

The DBSI metric of cellularity revealed significant differences between the non-inflammation and inflammation placentas, whereas the standard diffusion tensor imaging metric of ADC did not differentiate between the two groups. This difference is likely because diffusion tensor imaging cannot distinguish different diffusion components within an imaging voxel, so ADC reflects the average water diffusion across all placental microstructure components within the same voxel. In contrast, DBSI can specifically detect diffusion of water molecules within cells of a defined size range to reflect placenta immune cellularity. Specifically, the DBSI method can successfully image the immune cellularity in the human placenta by taking advantage of the fact that immune cells are generally much smaller than other cells in the placenta.

To strictly establish DBSI in accurately imaging and quantifying placenta immune cells, we conducted a careful validation study using the placenta collected in this study. Based on the RNA-sequencing results (Fig. 1F), we found the majority of placental immune cells were CD4 positive. We also conducted and tested other IHCs, including CD3, CD68, and CD79a, but none of them showed significant enough positive stains, similar to previously published studies^15,30^, so we did not include the data and results from other IHCs. Hence, we decided to validate the accuracy of the DBSI method by comparing it to IHC with an antibody specific to CD4. Our landmark-based voxel-to-region registration method allows accurate alignment between IHC and DBSI images, which enables quantitative, voxel-wise comparison between DBSI- and IHC-derived cellularity maps over the entire placenta tissue sections. In addition to IHC validation, our Monte Carlo computer simulation revealed that the DBSI method is accurate with signal-to-noise ratios as low as 25 dB on non-diffusion weighted b0 images. This is much lower than the typical signal-to-noise ratios of 50–75 dB in ex vivo MRI. Given our ex vivo and simulation data, we concluded that our DBSI method can sensitively and accurately image and quantify immune cell density in the placenta. Taken together, the validation measures all suggest that DBSI can accurately reflect in vivo inflammation in the placenta.

Our finding that inflammation significantly increased across pregnancy in the placental tissue compartment is consistent with previous reports that immune cells reside in the placental parenchyma and accumulate during pregnancy. Additionally, recent studies show that NK and T cells increase and differentiate in late gestation^31^, and neutrophils and macrophages infiltrate the placenta from the myometrium in late pregnancy to prepare for labor^32,33^. The immune cellularity in the placental vessel compartment was consistently low (around 5%) over pregnancy. Work from others suggests that immune cells in this compartment help to protect the fetus from infections^34^ and maintain uterine homeostasis^35^. The immune cellularity in the intervillous space was also consistently low (around 5%) over pregnancy. Mucosal-associated invariant T cells have been identified in the intervillous space and shown to accumulate upon bacteria stimulation^36^.

In this study, we are especially intrigued by our observation of localized cellularity in the inflammation placenta in Fig. 5D–F. On pathology, this placenta was noted to have a 2.5 cm cyst, but its location was not noted in the pathology report, so we were unable to determine whether it corresponded to the location of high cellularity. By conducting ex vivo DBSI of whole placentas in future studies, we may be able to identify DBSI features that reflect pathological observations.

In group-wise analysis, we found that immune cell density increased as pregnancy progressed in the non-inflammation group. This finding is consistent with previous observations that placental inflammation increases from second to third trimester^6,37^ and can trigger uterine contractions, cervical ripening and dilation, and placental separation^38–40^. Hence, placental inflammation has been suggested as a mechanism responsible for both term and preterm labor onset^41,42^. In future work, we will perform DBSI at more time points near the end of pregnancy to determine whether we can predict the onset of labor.

This study has serval limitations. First, immune cells have more complex geometries than the isotropic models we used in DBSI. As we defined any cell of radius less than 8 µm to be an immune cell, we could not assess the dynamic composition of different types of immune cells during pregnancy. In addition, relying solely on CD4+ cells as a marker for DBSI validation could overlook the presence of other immune cell types, potentially limiting the scope and comprehensiveness of the analysis. In future work, we can overcome these limitations by combining high-resolution immune cell 3D geometries with Monte Carlo simulations to develop more realistic diffusion signal models of various placental immune cells in DBSI. Additionally, in future studies, we plan to utilize more specific immune cell markers in IHC beyond CD4+ to enhance the accuracy and depth of the analysis. And we will also include more pathological data of patients for more comprehensive analysis. Second, we were unable to perform intra-placental segmentation in inflammation placentas in this study due to the significant motion of the fetus, uterus, and placenta during the imaging session. In future work, we aim to enroll more patients from both non-inflammation and inflammation placentas and conduct more high-quality compartment-based analyses, especially for the inflammation group, to determine whether particular regions of the placenta are more prone to immune cell infiltration based on the different pathologies behind it. Third, we did not account for potential confounders such as comorbidities, medication use, multiple gestations, and fetal anomalies in this study. In future research, we will incorporate these factors, as they may influence placental inflammation in human subjects.

## Methods

### Patient enrollment

This study was approved by the University’s Institutional Review Board, and all participants signed informed consent forms. Potential participants were included if they had a singleton pregnancy with no identified fetal anomalies at recruitment (before 12 weeks’ gestation) and intended to receive obstetric care and deliver at our institution. To make our study cohort homogenous, potential participants were excluded if they had multiple gestations, had a major fetal anomaly or had contraindications to MRI.

### In vivo MRI scans

MRI was performed on a 3 T Siemens Vida scanner (Siemens, Erlangen, Germany). Diffusion MRI of the entire uterus was acquired by a 2D EPI sequence in 86 different weightings (directions are the same as those in ex-vivo), with a max b-factor of 900 s/mm^2^. Eight b0 images were placed across the entire diffusion MRI scans. Other acquisition parameters were: 12.8 s repetition time, 62 ms echo time, in-plane resolution of 3 mm by 3 mm, and slice thickness of 3 mm. The total scan time was approximately 22 min. The Echo train of additional T2* MRI were: [3, 5.11, 7.2, 9.3, 11.4, 13.5, 15.6, 17.7, 19.8, 21.9, 24] (msec). The in-plane resolution was 4 mm by 4 mm, and the slice thickness was 4.2 mm. The FOV of Both T2* and diffusion MRI covers the entire placenta.

### Post delivery

This was a retrospective study, and we sorted the dMRI data into two groups based on post-delivery pathology. For all enrolled patients, the placenta was collected post-delivery for routine gross and histological exams performed by a board-certified pathologist with expertise in placental pathology (I.H.). Patients were divided into two groups (inflammation/non-inflammation) based on the findings on the pathology report: placenta noted to have villitis, chorioamnionitis, funisitis, chorionitis, or infarction and were defined as the inflammation cases. A subset (*N* = 13) of placenta samples were collected for single-nucleus RNA sequencing test. Placenta specimens were cut from a subset (*N* = 5) of placenta for ex-vivo validation between MRI and IHC and simulation validation.

### snRNA-seq

snRNA-seq was performed with 10X Genomics technology using the placental tissues collected in this study (www.10Xgenomics.com). Placental cell types and their relative percentages were annotated and visualized with t-distributed stochastic neighbor embedding (tSNE) (32), as presented in Fig. 2F. The size of variant types of cells was labeled, and the density of cell clusters was marked blue and red. Both cell size and density were correlated to the Diffusion Dictionary detected with diffusion basis spectrum imaging (DBSI).

### Ex-vivo validation: compare ex-vivo MRI and IHC

Specimens were collected from five fresh placentas after delivery to conduct ex vivo MRI scans. The samples were approximately 1 cm in width and length and were the entire height (thickness) of the placenta. Samples were stored at 4 °C, and within 12 h after delivery of the placenta, the specimens were embedded in a customized container filled with 2% agar. The ex vivo MRI was conducted on an 11.7 T Varian MRI scanner at 20 °C. A 2D EPI sequence was used, with TR = 1000 msec, TE = 32 msec, voxel size = 0.25 × 0.25 × 1 mm^3^, diffusion time = 16 msec, diffusion interval = 7 msec, 86 different diffusion weightings, and 5x diffusion intensity (b-max = 4500 s/mm^2^).

After MRI scans, the samples were bisected, fixed in 10% neutral-buffered formalin for one week, and processed into paraffin. Then, slide-mounted 5 µm histologic sections underwent immunohistochemistry with an anti-CD4 antibody (CONFIRM anti-CD4(SP35), Pre-dilute, Ventana, USA) and hematoxylin counterstain. Finally, the stained sections were scanned in a Hamamatsu NanoZoomer at 200x magnification. The details of quantification, co-registration, and correlation analysis between ex-vivo data can be found in Supplementary Material 2

### Diffusion basis spectrum imaging

A customized preprocessing pipeline for placental diffusion MRI data is shown in Supplementary Material 1. In DBSI analysis^26^, the diffusion MRI data are separated into a linear combination of signals across known physiological and pathological ranges (Eq. 1) (Fig. 2C). By solving the linear equation, the mixture of diffusion compartments inside a single imaging voxel can be revealed. The solved linear coefficients are the diffusion basis spectrum (Fig. 2D).

1 $${S}_{k}=\mathop{\sum }\limits_{i=1}^{{N}_{{aniso}}}{f}_{i}{e}^{-\left|\vec{{b}_{k}}\right|{\lambda }_{{\perp }_{i}}}{e}^{-\left|\vec{{b}_{k}}\right|\left({\lambda }_{{\parallel }_{i}}-{\lambda }_{{\perp }_{i}}\right){cos }^{2}{\phi }_{{ik}}}+\mathop{\sum }\limits_{j=1}^{{N}_{{iso}}}{f}_{j}{e}^{-\left|\vec{{b}_{k}}\right|{D}_{j}}$$

Where ${S}_{k}$ and $\vec{{b}_{k}}$ are the signal and *b*-value of the ${k}^{{th}}$ diffusion gradient; ${N}_{{aniso}}$ is the number of anisotropic tensors, ${\phi }_{{ik}}$ is the angle between the principal direction of the ${i}^{{th}}$ anisotropic tensor and the kth diffusion gradient; ${\lambda }_{{\parallel }_{i}}$ and ${\lambda }_{{\perp }_{i}}$ are the AD and RD of the ${i}^{{th}}$ anisotropic tensor, ${f}_{i}$ is the signal intensity fraction for the ${i}^{{th}}$ anisotropic tensor, ${f}_{j}$ is the signal intensity fraction for the ${j}^{{th}}$ isotropic tensor, and ${D}_{j}$ is the ADC of ${j}^{{th}}$ isotropic tensor. Additionally, conventional diffusion tensor imaging was performed to compute the ADC. The DBSI-derived ‘restricted’ isotropic diffusion fraction (*D*_*j*_ ≤ 0.6 μm^2^/ms) is defined to reflect cellularity ratio in placenta.

## Supplementary information

Supplementary Information

## Data Availability

The datasets used and analyzed during the current study are available from the corresponding author on reasonable request.

## Code availability

The underlying code for this study is not publicly available but may be made available to qualified researchers at a reasonable request from the corresponding author.
