# Supplementary material for: Quantitative and longitudinal assessment of human placental inflammation using diffusion basis spectrum imaging
Source: NPJ Womens Health. 2025 Jan 3;3(1):1. doi: 10.1038/s44294-024-00049-5 (PMC11698687; doi:10.1038/s44294-024-00049-5)
Supplement: Supplementary file 1 — Supplementary Information [file 44294_2024_49_MOESM1_ESM.pdf]

Supplementary Material

Supplementary Material 1: Preprocessing and Visualization

We first conducted the intra-diffusion weighting registration (**Supplementary Figure 1**). The raw data associated with each diffusion weighting was grouped into odd and even volumes (extract the odd/even slices). Then, the even volume was registered to the interpolated volume at the location of even slices. Finally, we finished the intra-diffusion weighting registration by merging the registered even volume with the original odd volume. This process was repeated on the raw data associated with every diffusion weighting. To further overcome the misalignment in inter-diffusion weighting, we developed a registration pipeline designed for placental dMRI. All diffusion-weighted volumes were registered to the first b0 image volume. We found the crop in pre-processing allows the registration to focus on the misalignment in the placenta region and saves computation time. However, both automatic intra-volume and inter-volume registration cannot guarantee to fix the misalignment in the fetal region, since the fetal movement can be way more complex as it possibly involves head turning, body flipping, and limbs movement. Hence, we conducted a visual check and further tuned parameters on the volumes that failed to converge. Also, in both registrations, we used a 0.25 sampling rate in computing similarity metric to save the overall computation time.

Although we didn't find any research on placenta dMRI that mentioned the signal drift issue, the phenomenon was reported in brain dMRI studies before. The phenomenon resulted from system thermal decay with failing fat suppression over time, especially here we had a long scan time as a long b-table was designed. Our in-vivo and ex-vivo results also showed the necessity of signal drift correction.

Supplementary Material 2: Ex-vivo Validation Pipeline

To align the 2D histology image and the 3D MRI volume, structures that were visible in both were labeled and used as landmarks (**Supplementary Figure 2 A, C**). Because landmarks on MRI are 3D and those in histology are 2D, the 2D MRI slices were first reconstructed on a curved plane (**Supplementary Figure 2 B**), defined by the landmarks. Next, the correspondence between one voxel on the MRI and a region on the raw histology image was computed (**Supplementary Figure 2 D-F**). The regions were typically 300 x 300 voxels, though this could vary because of deformation in landmark registration. For each MRI voxel, the ratio of cellularity area over total area was computed to generate the cellularity map. Finally, with the DBSI- and IHC-derived density maps aligned, the average values in 5-by-5 blocks of voxels were determined (**Supplementary Figure 2 G, H**). Values were analyzed in scatter plots with linear regressions (**Supplementary Figure 2 I**).

Supplementary Material 3: Additional Monte-Carlo simulations.

We computed the ADC of simulated diffusion MR signal from water molecules inside sphere models with increasing diameters, from 1 to 60 um (**Supplementary Figure 3 A–B**). The simulation was conducted using the same diffusion time, diffusion interval, and b-table. From the correspondence between diameter and ADC, we conclude that [16.3 μm, 0.6 μm/msec] is the desired threshold for in-vivo dMRI at 37 °C, [17.2 μm, 0.4 μm/msec] is the desired threshold for ex-vivo dMRI at 20 °C.

We compared the estimated cell ratio with and without signal drift at different ground truth cell ratios. In both linear signal drift and quadratic signal drift, the estimated cell ratios are remarkably decreased after simulated signal drift is applied. (Linear: **Supplementary Figure 3 C–D**; Quadratic: **Supplementary Figure 3 E–F**).

We built mixtures of multiple random sphere/ellipsoid models to produce the simulated dMRI signal, with a ground truth cellularity ratio ranging from 0 to 50%. The estimated cellularity ratio is heavily affected by signal drift (red scatters in **Supplementary Figure 3 D, F**)

Supplementary Material 4: Geometrical model based on histology and simulation validation

An automatic pipeline was built to convert a CD4-stained histologic image (**Supplementary Figure 3 A**) to a 3D model (**Supplementary Figure 3 G**) compatible with Monte Carlo simulation. Spheres with radius of 4–8 μm were used to model the immune cells in CD4+ areas (**Supplementary Figure 3 E**). A double-layer membrane structure was used to model the syncytiotrophoblast on the boundary of terminal villi (**Supplementary Figure 3 C**). Given the lack of cell boundary information on the CD4 staining, the rest of the regions were filled with hexagonal prisms (**Supplementary Figure 3 B**). Random seeds representing water molecules were placed in the spheres to simulate diffusion in immune cells (**Supplementary Figure 3 E**), in the double-layer membrane structure to simulate diffusion in the syncytiotrophoblast (**Supplementary Figure 3 C**), in hexagonal prisms to simulate diffusion in placental stromal cells (**Supplementary Figure 3 B**), and outside the model (**Supplementary Figure 3 D**) to simulate diffusion in intervillous spaces.

The same sequence parameters and b-tables as in the *ex vivo* scan were used in the simulation. The random diffusion of water molecules was simulated and recorded (**Supplementary Figure 3 F**). To collect the simulated signal, we summarized the signal decay of water molecules in each 0.25x0.25 region (**Supplementary Figure 3 G**) to obtain 400 simulated diffusion MRI signals with various signal-to-noise ratios.

Supplementary Material 5: Comparison of ADC in two groups showed no significant difference in four visits.

In parallel with DBSI analysis, we also present DTI analysis for reference. However, we have no positive finding to report on the longitudinal placental mean ADC value: No significant differences between the inflammation and non-inflammation groups. The two linear regressions look similar, with an overlapping 95% confidence range (**Supplementary Figure 4 C**). A comparison of each visit shows no significant differences (**Supplementary Figure 4 D-F**). Also, the ADC data looks more scattered (lower p and R^2 value).

Supplementary Figures

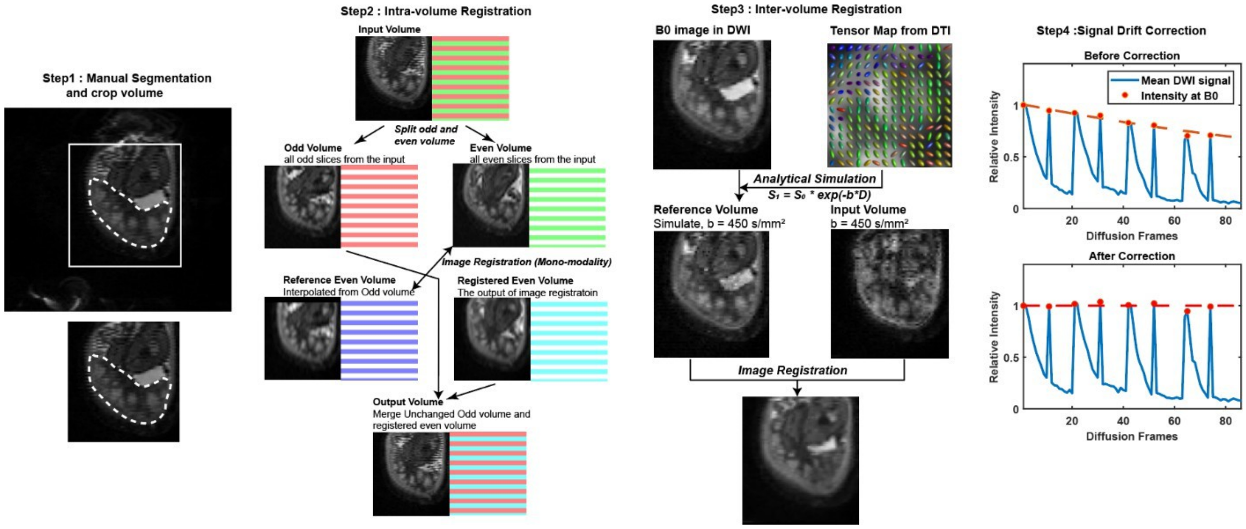

Supplementary Figure 1 Image preprocessing: The raw MRI were preprocessed before diffusion analysis (DTI & DBSI), including 1) volumetric crop centered at placenta region; 2) Intra-volume registration; 3) Inter-volume registration; 4) Signal drift correction.

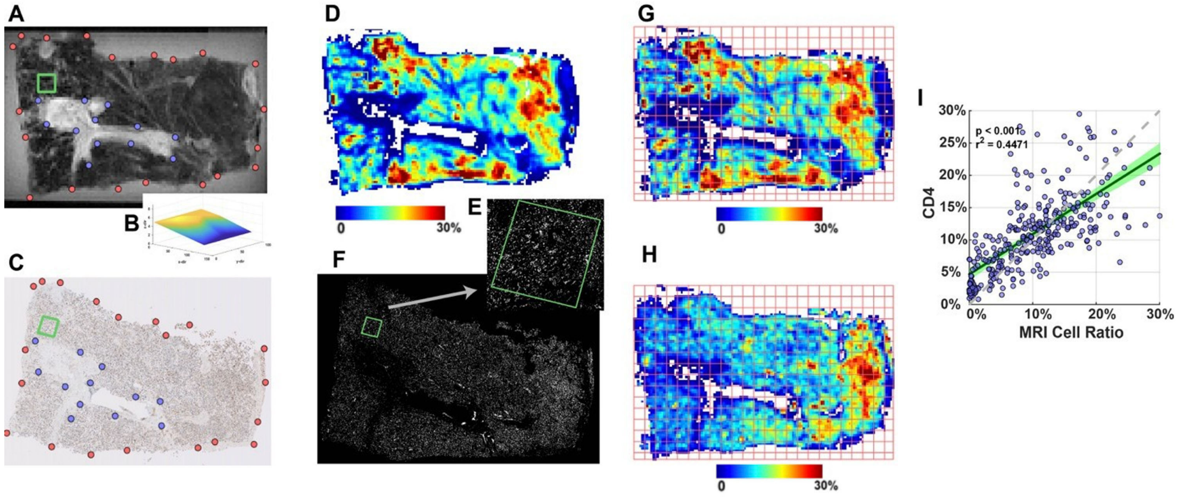

Supplementary Figure 2 Ex-vivo validation pipeline: A) 2D curved reconstructed MRI image defined by the 3D landmarks on a b0 image. Red scatters were landmarks on the boundary, blue scatters were landmarks inside the specimen, and a selected region was marked in a green box. B) Curved plan used for reconstruction. C) CD4 staining raw data, landmarks denoted in the same format as in A, the corresponding region on histology image marked in a green box. D) Maps of DBSI-derived immune cell density. E) Zoomed-in view of the positive stain area segmentation corresponding to the selected MRI region. F) Full map of the positive stain segmentation on CD4 IHC image. G–H) The MRI-derived and IHC-derived immune cell density maps were divided into 5-by-5 blocks of pixels for correlation tests. Only the blocks containing more than 5 non-zero values were included in the correlation. I) Each dot represents the average value in G) and H). P-value, R^2 value, and 95% confidence range from linear regression are presented.

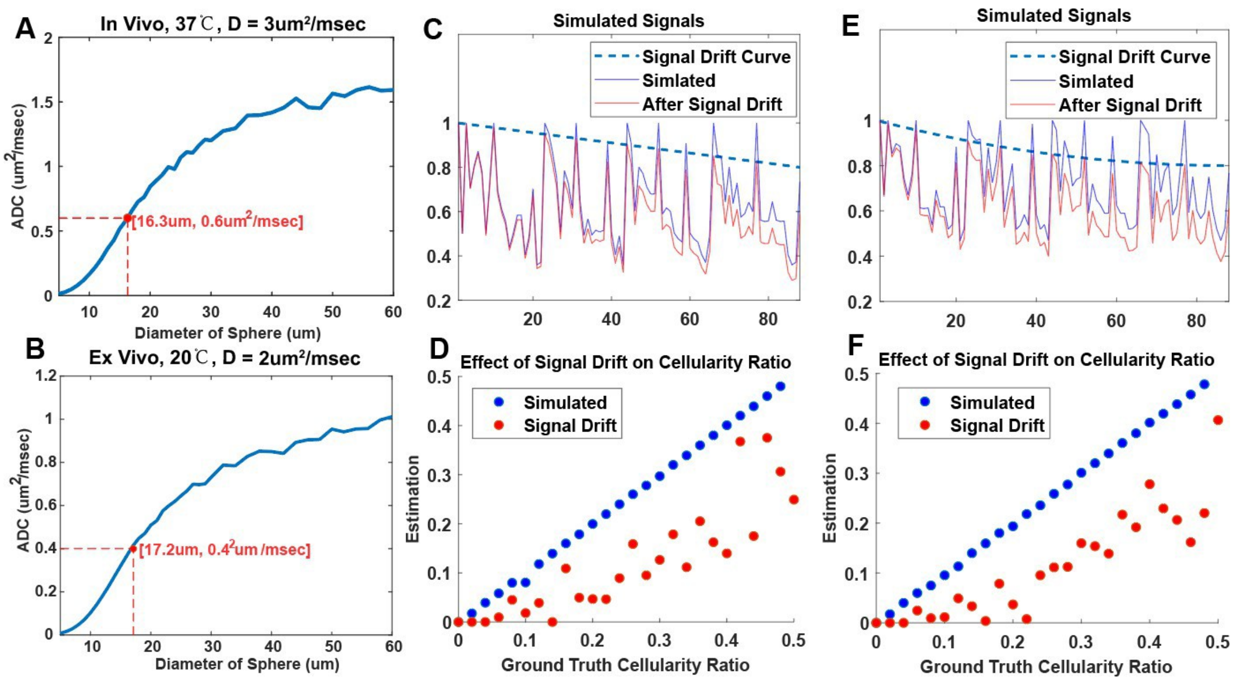

Supplementary Figure 3. Monte-Carlo validations of ADC thresholding and signal drift correction. See the details in Supplementary Material 3

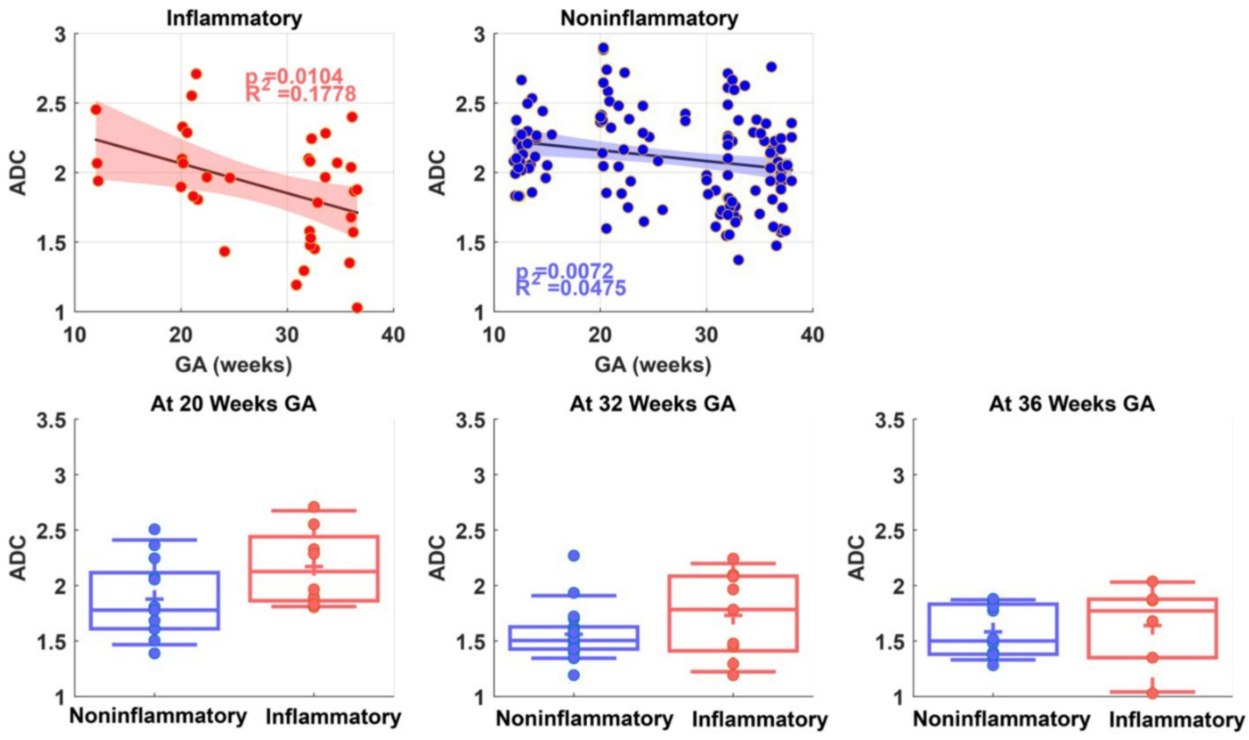

Supplementary Figure 4: Longitudinal placental DTI-ADC in two groups: A) Mean placental ADC in the inflammation group. B) Mean placental ADC in the non-inflammation group. C) Combined plot of A) and B). D–F) Comparison of mean placental ADC between groups in each visit.

Supplementary Table 1: Statistical analysis and detailed Information of 12 patients in the inflammation group.

Statistical analysis on key characteristics				
	Age	BMI	Gestational Age	Mode of delivery
Non-inflammation Group	29.28 ± 4.98	28.04 ± 5.79	266.58 ± 20.62	Spontaneous vaginal (73%) and non-spontaneous vaginal (27%)
Inflammation Group	29.17 ± 4.15	27.42 ± 5.89	265.83 ± 18.12	Spontaneous vaginal (83%) and non-spontaneous vaginal (17%)
p-value	0.88	0.76	0.90	0.72
Significant difference	No	No	No	No

Patient #1			
Age: 36	BMI: 23.16	Pregnancy History: G2P1001	Delivery: Live male @40w2d, Apgar 8/9
Medical History: 1. Prothrombin mutation 2. Anemia			
Placenta Histology: 1.Basal plate myometrial fibers 2. **Multifocal villitis with associated intervillositis** 3. Changes suggestive of stem vessel obliteration 4. **Fetal membrane with pigmented amniotic macrophages**.			

Patient #7			
Age: 31	BMI: 28.34	Pregnancy History: G2P0100	Delivery: Live male @39w1d, Apgar 7/9
Medical History: 1. History of prior intrauterine fetal demise, 2. Shoulder dystocia, 3. IUGR in this pregnancy			
Placenta Histology: 1.**Fetal membranes with numerous pigment-laden amniotic macrophages** 2. Focal increase in preivillous fiberinoid deposition 3.marginal cord insertion			

Patient #14			
Age: 20	BMI: 18.54	Pregnancy History:G2P1001	Delivery: live female @40w2d, Apgar 8/9
Medical History: 1. first trimester RPR was reactive, third trimester RPR was nonreactive 2.HSV+/bright light exam- 3. positive Trichomonas. 4.Asthma, 5.Depression, 6.Anemia, 7.Gonorrhea,8.Chlamydia,9.Syphills,10. UTI			
Placenta Histology: 1.**Fetal membrane with moderate acute chorioamnionitis** 2. **Trivascular cord with modera funisitis** 3. Small disc <10th percentile 4. Delayed villous maturation.			

Patient #35			
Age: 27	BMI: 25.97	Pregnancy History: G4P2012	Delivery: Live male @37w5d, Apgar 7/9
Medical History: 1. Oligohydramnios 2. Increased LFT 3.History of PEx2 4.Cholecystitis 5. Acute kidney injury 6. Right UPJ obstruction 7.GBS positive			

Placenta Histology:
**1. Acute chorionitis**<br>**2. Trivascular cord with umbilical arteritis**

Patient #42			
Age: 26	BMI: 33.80	Pregnancy History:G5P4004	Delivery: Live male @36w4d, Apgar 8/9
Medical History: 1. Depression 2. Gestational thrombocytopenia @39wk5d GA in pervious pregnancies			
Placenta Histology:<br>1. **Fetal membranes with numerous pigmented amnionic macrophages**<br>2. Histologically unremarkable three-vessel cord<br>3. **Chronic lymphohistiocytic villitis, non-necrotizing**			

Patient #44			
Age: 29	BMI: 28.30	Pregnancy History: G3P1011	Delivery: Live male @39w5d , Apgar 8/9
Medical History: 1. History of anemia			
Placenta Histology:<br>1. **Fetal membranes with pigmented amniotic macrophages**<br>2. Delayed villous maturation<br>3. **Chronic lymphohistiocytic villitis**<br>4. **Increased perivillous fiberinoid deposition, approximately 10% of volume**			

Patient #48			
Age: 32	BMI: 22.04	Pregnancy History:G5P2022	Delivery: Live female @37w1d, Apgar 8/8
Medical History: 1. Trichomoniasis, 2. Bacterial Vaginosis, 3. Group B Strep positive, 4. Yeast infection, 5. Gestational diabetes in this pregnancy.			
Placenta Histology:<br>1. **Chronic lymphohistiocytic villitis, mild/focal, non-necrotizing**<br>2. Small disc, <10th percentile			

Patient #50			
Age: 29	BMI: 35.98	Pregnancy History: G2P0101	Delivery: Live male @34w5d, Apgar 6/8
Medical History: 1. Chronic hypertension, 2.Depression			
Placenta Histology:<br>1. **Fetal membranes with pigmented amniotic macrophages**<br>2. Multiple intervillous throbohematomas<br>3. **Acute chorionitis**			

Patient #60			
Age: 27	BMI: 20.45	Pregnancy History: G3P0111	Delivery: Live male @ 32w0d, Apgar 8/9
Medical History: 1.History of opioid use, and methadone daily. 2. Short cervix and asthma			
Placenta Histology:<br><br>1. **Fetal membrane with acute chorioamnionitis**<br>2. Small disk <10th percentile			

Patient #109			
Age: 34	BMI: 25.14	Pregnancy History: G4P2012	Delivery: Live male @37w5d, Apgar 8/9
Medical History: 1. History of PE with severe features, 2. Anaplastic ependymoma			
Placenta Histology:<br><br>1. **Incidental septal cyst (2cm in greatest dimension)**<br>2. Infarct (1.5cm in greatest dimension)			

Patient #112			
Age: 28	BMI: 32.01	Pregnancy History: G3P2002	Delivery: Live male @ 39w1d, Apgar 8/9
Medical History: 1. Anemia 2. History of low transverse cesarean section, 3. Group B strep positive.			
Placenta Histology:<br><br>3. **Intervillous fibrin**<br>4. **Peripheral placental cyst with 0.9 cm greatest dimension.**			

Patient #116			
Age: 31	BMI: 35.35	Pregnancy History: G1P1000	Delivery: Live male @39w4d, Apgar 7/9
Medical History: 1. History of gestational hypertension.			
Placenta Histology:<br><br>1. **Fetal membranes with pigmented amniotic macrophages**<br>2. **Mild acute chorioamnionitis**			
